# Detection of internal exon deletion with exon Del

**DOI:** 10.1186/1471-2105-15-332

**Published:** 2014-10-16

**Authors:** Yan Guo, Shilin Zhao, Brian D Lehmann, Quanhu Sheng, Timothy M Shaver, Thomas P Stricker, Jennifer A Pietenpol, Yu Shyr

**Affiliations:** Vanderbilt Ingram Cancer Center, Center for Quantitative Sciences, 2220 Pierce Ave, 549 Preston Research Building, Nashville, TN 37232 USA; Department of Biochemistry, Vanderbilt University, Nashville, TN 37232 USA; Department of Pathology, Vanderbilt University, Nashville, TN 37232 USA

## Abstract

**Background:**

Exome sequencing allows researchers to study the human genome in unprecedented detail. Among the many types of variants detectable through exome sequencing, one of the most over looked types of mutation is internal deletion of exons. Internal exon deletions are the absence of consecutive exons in a gene. Such deletions have potentially significant biological meaning, and they are often too short to be considered copy number variation. Therefore, to the need for efficient detection of such deletions using exome sequencing data exists.

**Results:**

We present ExonDel, a tool specially designed to detect homozygous exon deletions efficiently. We tested ExonDel on exome sequencing data generated from 16 breast cancer cell lines and identified both novel and known IEDs. Subsequently, we verified our findings using RNAseq and PCR technologies. Further comparisons with multiple sequencing-based CNV tools showed that ExonDel is capable of detecting unique IEDs not found by other CNV tools.

**Conclusions:**

ExonDel is an efficient way to screen for novel and known IEDs using exome sequencing data. ExonDel and its source code can be downloaded freely at https://github.com/slzhao/ExonDel.

**Electronic supplementary material:**

The online version of this article (doi:10.1186/1471-2105-15-332) contains supplementary material, which is available to authorized users.

## Background

Exome sequencing is one of the most cost-efficient sequencing approaches for conducting genome research on coding regions. The primary applications of exome sequencing include detection of single nucleotide polymorphisms, somatic mutations, small and large structural variations, and copy number variations. There are also some less obvious data mining opportunities through exome sequencing data such as extraction of mitochondrial [1] and viral sequences [2]. Another less explored genomic aberration that can be detected through exome sequencing is internal exon deletions (IEDs). Not to confuse with exon skipping, IEDs are the result of the deletion of one or more consecutive exons in a gene where exon skipping are artificial method used to encourage the cellular machinery to skip over an exon [3].

Functional IEDs were first described in murine T-cell acute lymphoblastic leukemia (T-ALL), in which constitutive ligand-independent activation of NOTCH1 occurs from a deletion of exons 3-27, preserving the transcriptional binding domain in exons 28-34 [4]. A similar IED was recently reported in a breast cancer cell line, HCC1599 [5]. The number of deleted exons range from a single exon to nearly the whole gene as in the example of the HCC1599 cell line. These IEDs are often too short to be considered copy number variation, thus only the very large ones have a chance to be picked up by sequencing-based CNV detectors. IEDs have biological importance in cancer, such as in the removal of important regulatory mechanisms or protein-protein interaction domains. Given the large amount of publically available exome sequencing data accumulated over the last few years, a method that can efficiently detect such deletions would benefit the medical research community greatly and provide means to rapidly identify new IED candidates. Thus, we have designed ExonDel, a tool aimed at detecting IEDs through exome sequencing data. ExonDel is written in a combination of Perl and R. ExonDel detects exon deletion at gene level rather than at global level, and it adjusts for GC content.

## Implementation

An IED can be homozygous or heterozygous. While homozygous means that exon is deleted in both allele and heterozygous means that the exon is deleted in one of the two alleles. Homozygous deletion is relatively easier and more accurately detectable than heterozygous exon deletion. ExonDel is currently designed to detect homozygous IEDs only. ExonDel differs from other sequencing-based CNV tools by detecting exon deletion on a per gene level instead of searching for large lengths of depth variation across the whole genome. To achieve this, ExonDel first computes callable genes based on different exome capture methodologies. There are three major exome sequencing capture kits currently in broad use: Illumina TruSeq, Agilent SureSelect, and NimbleGen SeqCap EZ. The target regions for these three exome capture kits vary and range from 37.6 to 62.1 million base pairs. The capture kits available can enrich the exome, and additional content includes exons plus 3’ and 5’ UTRs. The capture kits differ in their target regions, bait length, bait density, and molecule used for capture. To account for these differences, ExonDel computes the callable genes first. A callable gene has to satisfy the following two conditions: 1) all exons of this gene must be covered by the exome capture kit, and 2) each exon must have at least 90% of its base pairs covered by the exome capture kit. The first condition ensures that no false positive resulted from uncovered exons in the capture kit. The second condition ensures that no false positives resulted from partially covered exons in the capture kit. ExonDel will only attempt to detect exon deletions for the callable genes.

The important inputs of ExonDel include a non-optional Binary Alignment Map (BAM) [6] file, a non-optional Browser Extensible Data (BED) file of the capture kit, and an optional Gene Feature Format (GTF) file. The BED file provides the exact capture regions down to a single base-pair resolution. The GTF file provides detailed information about the starts and ends of exons. Both BED and GTF files are used to compute callable genes: if GTF is not provided, ExonDel will apply the latest gene annotation from RefSeq. Other input parameters of ExonDel include a maximum window size and a list of genes of interest. The maximum window size parameter determines the max length IED that ExonDel will search for. For example, if the maximum window size is 7, ExonDel will search for IEDs with length less than 7 exons. For the user-input list of genes of interest, instead of searching though the entire exome, ExonDel will only search IEDs in the genes of interest in order to save time.

**Figure 1:**
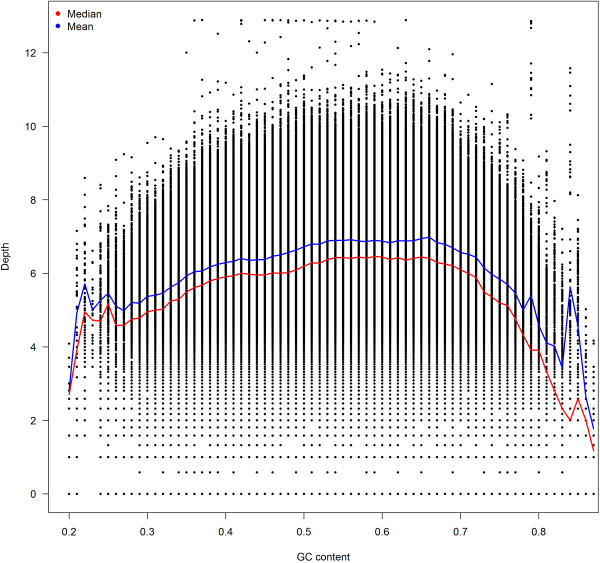
**Using data from all 16 samples, we show that depth drops for exons with low and high GC content.**

The depth coverage of Illumina sequencing data can be influenced by GC content [7]. Many sequencing-based tools have taken the GC content’s effect on depth into consideration. We also observed similar bias based on analysis of exon depth (Figure 1). To minimize the effect of GC-content bias on depth, we applied standardization of depth by GC content followed by median correction, a method described in [8]. The GC content was adjusted by the following formula,  where r_*i*_ are the read counts of the *ith* exon, and *m*_GC_ is the median read counts of all exons that have the same GC content as the *ith* exon. In Yoon et al.’s original implementation, *ri* is the read counts of the *ith* 100 bp window because copy number was under consideration instead of exons.

ExonDel detects exon deletions by comparing each exon’s depth against its parent gene’s median depth after performing the depth adjustment by GC content described previously. To ensure high specificity, reads with poor mapping quality (MQ < 20 for BWA aligned BAMs) are removed. If non BWA aligned BAMs are used (such as BAMs from Bowtie 2 [9], where mapping quality definition is different), ExonDel will compute the average base quality per read as  where *l* is the length of the read, and *BQi* is the base quality of *ith* nucleotide. All reads with average *BQ*_*r*_ < 20 are removed.

IEDs can have lengths 1 to L-1, where L is the total number of exons in a gene. Longer IEDs are more reliable than shorter IEDs because shorter IEDs, especially for IEDs with length 1 are more likely to be false positives caused by sequencing artifacts. Potential IEDs will be identified if the median depth of one or consecutive exons are smaller than a certain user-adjustable percentage of the median depth within the gene. First we define the median depth of the *ith* exon as *DP*_*e*_*i* = *median* (*DP*_1_*to DP*_*l*_), where *l* is the length of the exon. The list of all median depths of all exons is denoted as *DP*_*all*_. An exon is considered deleted if and only if the following conditions are satisfied:

1. *DP*_*e*_*i* < *C*1 % × *DP* _*all*_, *C*% × *DP all* presents the percentile of all exon depth, and *C* is a constant. By default, *C* is 2. The user can manually adjust *C* to change the sensitivity of ExonDel. Increasing *C* will result more IEDs detected.
2. *DP*_*ē*_ > *C2* % *Î§ DPall*, where *ē* denotes the exons that do not satisfy condition 1. By default *C* is 10.

ExonDel performs the exon deletion detection at gene level. Once it moves to the next gene, the condition is reset. The number of exon deletions detected is inversely proportional to this parameter (Table 1). When multiple samples are loaded, ExonDel computes the summary statistics of all samples.

**Table 1:** **Exon deletion candidates identified by window length, and comparison with other CNV tools using breast cancer cell lines**

Deletion window length	1	2	3	4	5	6	7	8	9
Exon Deletions^1^	13720	163	23	11	9	8	8	6	6
Verified in RNAseq^2^	1988	17	7	7	7	6	6	6	6
Found by CNV Tools^3^	6099	129	20	11	9	8	8	6	6

^1^Based on 16 samples.

^2^Based on 13 samples, 3 of the 16 samples did not have RNAseq data.

^3^Based on 16 samples.

To demonstrate the effectiveness of ExonDel, we used two independent datasets. The first dataset contains exome sequencing data from 16 breast cancer cell lines. The exomes were captured using Illumina’s TrueSeq capture kit. Seventy five nucleotide paired-end sequencing was performed using Illumina’s HiSeq 2000 platform at Vanderbilt Genomic Core. RNAseq data RT-PCR were used to validate the IEDs identified by ExonDel. Because ExonDel is also designed to work with tumors, which are heterogeneous (a mixture of tumor and normal tissues) compared to cell line, we downloaded exome sequencing data of 10 breast cancer tumor samples ("TCGA-A7-A0D9", "TCGA-BH-A0B3", "TCGA-BH-A0B8", "TCGA-BH-A0BJ", "TCGA-BH-A0BM", "TCGA-BH-A0C0", "TCA-BH-A0DK", "TCGA-BH-A0DP", "TCGA-BH-A0E0", "TCGA-BH-A0H7") from The Cancer Genome Atlas (TCGA). The corresponding RNAseq data of the same 10 samples were also downloaded for validation purpose.

## Results

**Figure 2:**
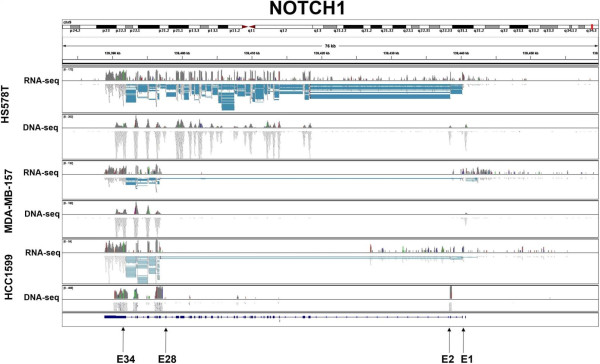
**Verification of NOTCH1 deletions found by ExonDel.** The mapping results of both exome sequencing and RNAseq data support the large deletion in NOTCH1. RNAseq data further proves that such deletions can be carried through transcription to RNA.

**Figure 3:**
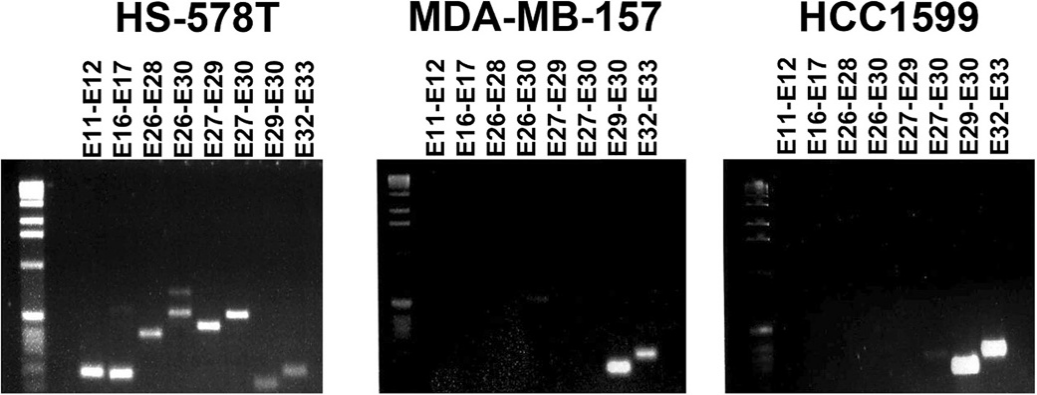
**Further validation of exon deletion on NOTCH1 was obtained using RT-PCR.**

The 16 cell line datasets were processed in house using standard sequencing processing pipeline. The complete raw quality control results can be seen in Additional file 1: Table S1. Alignment was done using BWA [10] against the HG19 human genome reference. Statistics of alignments can be viewed in Additional file 2: Table S2. Using ExonDel to screen for IED on the 16 cell line samples, we identified both novel and known exon deletions were observed. We were able to validate the previously described deletion of exons 3 to 27 in NOTCH1 in cell line HCC1599 and identify a similar deletion in cell line MDA-MB-157. This previously unidentified IED of exons 2 to 27 is similar to the deletion in HCC1599 and those described in murine T-ALL [4, 5]. To verify these findings we performed RNAseq on these cell lines. Figure 2 depicts the sequencing depth coverage using Integrative Genomics Viewer for both DNA exome and RNAseq data for each of the cell lines. For comparison, we included a cell line without the NOTCH1 deletion (HS578T) in Figure 2. Exons 3-27 and 2-27 are clearly deleted in HCC1599 and MDA-MB-157 respectively but remain intact in HS578T cells. These deletions are further confirmed with RT-PCR (Figure 3).

**Figure 4:**
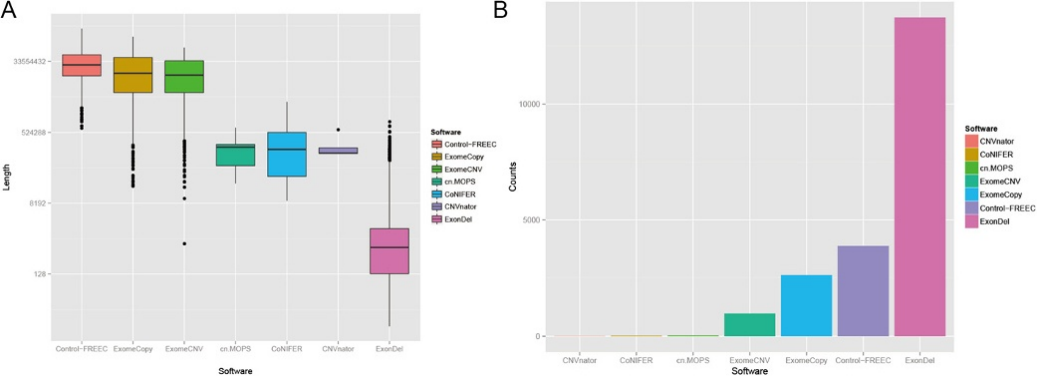
**Comparison between ExonDel and other CNV calling tools.** **(A)** The length distribution of the deletion detected by all tools; **(B)** The number of deletions detected using all tools at window size 1.

In addition to the multi-exon deletion in NOTCH1, many novel IED candidates were identified, some containing as few as a single exon (Table 1). IEDs with a single exon are more likely to result from sequencing artifacts. For comparative purposes, we screened our samples for CNV using 6 sequencing data-based CNV callers: ExomeCNV [11], CNVnator [12], CoNIFER [13], Control-FREEC [14], ExomeCopy [15] and cn.MOPS [16]. Even after combining results from all six CNV tools, ExonDel can still indentify many novel deletion candidates not identified by other CNV tools (Table 1). Figure 4A demonstrates the distribution of length of deletions detected by each tool. Clearly, ExonDel can identify smaller deletions while other CNV tools identified deletions with long length. Figure 4B shows the number of deletions detected by each tool. At window size 1, a significantly more number of potential IEDs were identified by ExonDel on all 16 samples, given researcher a greater chance at identifying the true biological relevant IDEs. The NOTCH1 deletion we described was identified in 3 out of the 6 tested CNV tools. The detailed results of the ExonDel and CNV comparison can be viewed in Additional file 3: Table S3.

We repeated the exon deletion analyses on the TCGA tumor datasets. Because we do not have access to the actual tumor sample, we could not perform RT-PCR validation. RNAseq data was used for validation and comparisons with the six CNV tools were conducted. Results of similar patterns were identified. More IEDs can be detected with smaller window size, and ExonDel was able to find more IEDs at all windows sizes compared to the other six CNV tools combined (Table 2). This result shows that ExonDel is able to perform well on tumor samples.

**Table 2:** **Exon deletion candidates identified by window length, and comparison with other CNV tools using the 10 TCGA breast cancer tumor samples**

Deletion window length	1	2	3	4	5	6	7	8	9
Exon deletions	13494	1734	524	230	94	38	20	13	7
Verified in RNAseq	4722	489	174	70	36	16	10	7	4
Found by CNV tools	2635	584	268	154	73	33	17	12	6

## Discussion

IEDs have functional implications in cancer genomics and we have developed a tool, ExonDel, to screen for novel IED candidates efficiently. Using a combination of Perl and R, we provide a single package including all source codes and instructions which is freely available for download. While providing several important new features, ExonDel also contains a few limitations. For example, as the name indicates, ExonDel can only detect exon deletion not amplification. The window size plays a significant role in detection of IED. Large window size ensures more accurate detection at the cost of missing small IEDs. Small window size on the other hand, allows to detection high number of IEDs at the cost of higher false positive rate. Thus, we recommend running ExonDel at window size 1 to 7 in one setting, and scan for potential biological meaningful IED candidates from the results of larger window size to smaller window size.

ExonDel distinguish itself from other sequencing-based CNV tools in two aspects. First, it performs deletion detection at gene level and uses exon as unit. Other sequencing-based CNV tools usually consider CNVs as large deletion or duplication spanning large genomic regions. It is common to see that CNV contains many genes and the median length of CNV detected using sequencing-based CNV tool is around 10^5^ [17] and the average exon less is less than 200 base pairs [18]. Thus, ExonDel is very efficient, and one exome can be screened in about 15 minutes. ExonDel also allows the user to define the deletion window size and can be configured to run multiple BAM files in parallel.

In theory, ExonDel is designed to work with both tumor and cell line samples. Tumor samples differ from cell lines samples because they are usually a mixture of tumor and normal tissues. Thus, tumor sample contains noises which can mask the true variant signal. This is a challenge all variant callers have to face. If the tumor purity is low, a deleted exon might have reads aligned to it due to the presents of normal tissue. In such cases, ExonDel would not able to identify such IEDs. As shown in our TCGA tumor dataset results (Table 2), ExonDel was able to identify many potential IEDs, and a significant portion of them were verifiable by RNAseq and other CNV tools. This indicates that either the purities of these tumors were good, or many true IEDs were not affected by tumor heterogeneity. A portion of IEDs might still be affected by tumor heterogeneity, and these IEDs were not detectable by ExonDel.

## Conclusion

Given the large volume of exome sequencing data publically available in repositories such as TCGA, the 1000 Genomes Project, NHLBI Exome Sequencing Project, and The Sequence Reads Archive, ExonDel provides researchers with a powerful tool to mine for internal deletions that may contain novel biological findings.

## Availability and requirements

**Project name:** e.g. ExonDel project

**Project home page:** e.g. https://github.com/slzhao/ExonDel

**Operating system(s):** Linux

**Programming language:** Perl, R

**License:** GPL v2

**Any restrictions to use by non-academics:** No

## Electronic supplementary material

Additional file 1: **Raw data quality control matrix.** (XLSX 13 KB)

Additional file 2: **Alignment quality control matrix.** (XLSX 15 KB)

Additional file 3: **Comparison between ExonDel and six other CNV tools.** (XLSX 2 MB)
